# The effect of experimentally induced acute pain on lumbar movement control: a single blinded three-arm cross-over randomized control trial

**DOI:** 10.1186/s12891-026-09847-1

**Published:** 2026-04-18

**Authors:** Beate Schüßler, Gabriela Ferreira Carvalho, Tibor Maximilian Szikszay, Patrizia Khan, Stefan Sebastian Niemuth, Carla Nau, Kerstin Luedtke

**Affiliations:** 1 Department of Physiotherapy, Institute of Health Sciences, Pain and Exercise Research Luebeck (P.E.R.L.), University of Lübeck, Lübeck, Germany; 2 Department of Physiotherapy, Faculty of Health, Medical & Life Sciences, Furtwangen University, Furtwangen, Germany; 3 Department of Anaesthesiology and Intensive Care, University Medical Center Schleswig-Holstein, Campus Lübeck and University of Lübeck, Lübeck, Germany

**Keywords:** Low back pain, Movement control, Pain mechanisms, Experimental pain, Acute pain

## Abstract

**Background:**

Non-specific low back pain exhibits high prevalence and is commonly correlated with deficits in lumbar movement control. Whether pain precipitates these impairments or arises as their sequela remains uncertain. This study investigates how experimentally induced acute pain influences lumbar movement control.

**Method:**

Forty-five healthy, pain-free participants underwent three experimental conditions in randomized order: hypertonic saline injection to the lumbar paraspinal muscle (inducing local pain), hypertonic saline injection to the deltoid muscle (inducing remote pain), and isotonic saline injection to the lumbar paraspinal muscle (sham). A standardized, reliable and validated battery of lumbar movement control tests was performed before, during and after each condition and rated by an examiner blinded towards the experimental intervention. Perceived pain intensity was evaluated every 30 s using the numeric rating scale.

**Results:**

While initial analysis revealed a significant overall effect (χ^2^(8) = 23.45; *p =* 0.003, W = 0.26), post hoc pairwise comparisons showed no significant differences of movement control test results either within or between experimental conditions. Mean differences following the injections were small (pain_LB_ vs sham: MD -0.29, 95% CI -0.70 – 0.12; pain_ARM_ vs sham: MD -0.16, 95% CI -0.56 – 0.25; pain_LB_ vs pain_ARM_: MD 0.07, 95% CI -0.41 – 0.55). As expected, pain intensity was significantly lower in the sham condition than in either experimental condition, confirming successful pain induction. Exploratory regression analyses revealed no significant associations between pain intensity on lumbar movement control.

**Conclusion:**

Experimentally induced acute pain exerted no detectable effect on lumbar movement control. Across analyses, performance remained stable, indicating short-term robustness to transient nociceptive input.

**Trial registration:**

DRKS00038877 (retrospectively registered; January 6th, 2026).

## Background

Low back pain is one of the most prevalent musculoskeletal conditions and a leading cause of disability worldwide, imposing substantial socioeconomic costs and reducing quality of life [1, 2]. Non-specific low back pain, classified as chronic primary musculoskeletal pain in ICD-11, refers to pain in the lower back persisting for at least three months without a clearly identifiable structural or pathoanatomical cause, and associated with functional impairment [3]. It accounts for the majority of low back pain cases but despite its prevalence, current treatment strategies often provide only moderate or short-term benefits, reflecting the heterogeneity of patient populations and limited understanding of underlying mechanisms [4–6]. Consequently, there is a need to move beyond approaches that primarily target pain relief or perceived discomfort and to investigate the neuromuscular and motor mechanisms that contribute to pain persistence and functional impairment.

Pain is known to induce immediate and longer-term adaptations in neuromuscular control [7, 8]. Acute nociceptive input can alter the recruitment and timing of deep trunk muscles, reduce movement variability, and lead to more rigid movement strategies, which serve as short-term protective mechanisms and but may also constrain sensorimotor feedback and movement efficiency [7]. Over time, repeated or persistent pain episodes can foster maladaptive patterns, including altered intermuscular coordination, delayed activation of stabilizing muscles, and cortical reorganization [9–12]. According to the theory proposed by Hodges and Tucker [8], these adaptations reflect a complex, non-linear interaction between pain, motor control and behavior, whereby changes in neuromuscular control are not only protective but also aim to maintain functional movement while minimizing pain.

Importantly, these neuromuscular adaptations are typically measured in experimental settings and may not always translate directly into clinically observable deficits. To capture functionally meaningful adaptations in a practical and clinically relevant manner, lumbar movement control has emerged as a construct that reflects how the neuromuscular system organizes task- and context-specific spine movements. Lumbar movement control refers to the ability to coordinate and regulate lumbar spine movements, minimizing excessive, premature, or compensatory movements while allowing efficient execution of functional tasks [13–15]. It integrates multiple dimensions of motor function, including spatial and temporal control, variability and adaptability of muscle recruitment, force modulation, and proprioceptive feedback, thereby reflecting the observable manifestation of underlying neuromuscular mechanisms such as muscle activation patterns, corticospinal excitability and sensorimotor integration [13, 14, 16]. Clinical assessments of lumbar movement control offer a practical method to detect subtle deficits that may not be apparent through strength testing or imaging, providing insight into the functional impact of pain on motor control.

Evidence indicates that impairments in lumbar movement control are common in individuals with chronic non-specific low back pain, with studies reporting that up to 80% of patients exhibit deficits in at least one movement direction distinguishing them from healthy individuals [17–20]. These impairments appear to progress as pain persists, implicating them in the transition from acute to chronic pain [11, 20–22]. Although acute or experimentally evoked pain can precipitate alterations in motor control, such as modified deep trunk muscle recruitment, delayed activation sequences, and reduced movement variability [7], it remains unclear whether these adaptations are detectable in clinical lumbar movement control assessments and to what extent they are reversible.

This gap is clinically and scientifically important: if acute nociceptive pain produces observable deficits in lumbar movement control, such changes could serve as early markers of maladaptive motor responses that predispose individuals to chronicity. Therefore, the present study aimed to investigate the effects of experimentally induced acute pain, both local and remote, on lumbar movement control, assessing whether pain leads to immediate and reversible changes in lumbar movement control. We hypothesized that local low back pain might impair lumbar movement control, whereas pain at a remote site would not significantly affect trunk movement. In addition, an exploratory analysis was conducted to examine whether demographic factors and pain intensity are associated with variability in lumbar movement control performance.

## Methods

### Study design

This single blinded three-arm cross-over randomized control trial was conducted at the University of Lübeck in adherence to the "Declaration of Helsinki" [23] and Consolidated Standards of Reporting Trials (CONSORT) guidelines [24]. Ethical approval was obtained (Ref: 22–114) the study was preregistered on the Open Science Framework (10.17605/OSF.IO/4APJW; October 15th, 2022) prior to study commencement and was retrospectively registered in the German Clinical Trials Register (DRKS; DRKS00038877; January 6th, 2026). No changes to the experimental procedures, outcomes, or analyses were made after the study commenced.

### Participants

The recruitment of participants took place from October 2022 to July 2023 at the University of Lübeck, the University Medical Center Schleswig-Holstein, and via social media. Healthy adults with no history of low back pain were included. Participants were required to speak German to fill out the questionnaires and to have full active knee and hip range of motion for unrestricted test performances. Exclusion criteria were: any pain report at baseline, any chronic pain longer than three months within the last two years, recent low back pain more than 24 h in the last month, diagnosed neurological, cardiovascular or psychological disease, a history of spinal surgery, regular medication use, pregnancy, breastfeeding, previous joint replacement surgery, inflammation or acute injury of the hip or knee joint, saline allergy and congenital or acquired coagulation disorders.

### Baseline characteristics and questionnaires

To describe the study population, basic demographic information (age, sex assigned at birth of participants, height, weight) and occupational posture (categorized as mainly) were assessed by self-report. Baseline psychological and behavioral characteristics were assessed using validated and widely used questionnaires. Fear of pain was measured using the Fear of Pain Questionnaire (FPQ), which assesses individuals’ fear responses to potentially painful situations [25, 26]. The FPQ was included to capture the general fear of pain. Pain-related fear of movement was assessed with the Tampa Scale of Kinesiophobia (TSK), a validated instrument measuring fear of movement and re-injury [27]. Physical activity levels and daily sitting time were assessed using the International Physical Activity Questionnaire – Short Form (IPAQ-S), which has demonstrated acceptable reliability and validity across multiple populations [28]. No study-specific questionnaires were developed for this research.

### Procedure

**Fig. 1:**
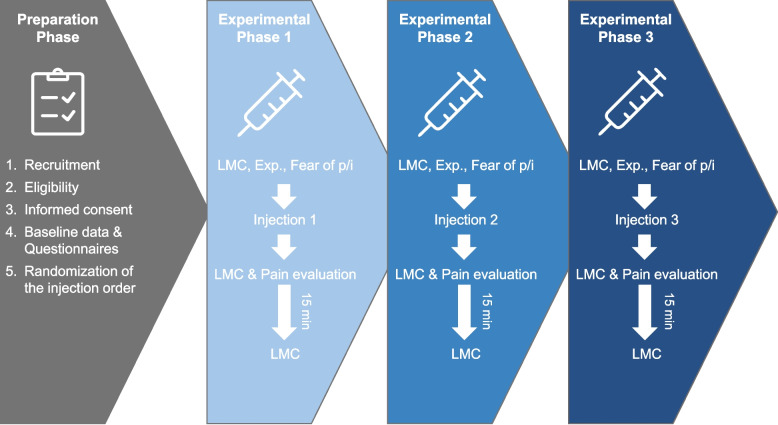
Study process: On the left the preparation phase, followed by the experimental phase which was conducted three times per participant under changing injection conditions in block wise randomized order: NaCl 5,85% (hypertonic saline) back or arm, NaCl 0,9% (isotonic saline) back; Questionnaires: International Physical Activity Questionnaire, Tampa Scale of Kinesiophobia, Fear of Pain Questionnaire; Pain Evaluation: Numeric Rating Scale (0–10) every 30 s, McGill Pain Questionnaire; (LMC = test battery lumbar movement control; p/i = pain/injection; min = minutes)

The study process consisted of two phases: the preparation phase and the experimental phase. The preparation phase involved recruiting participants, verifying eligibility, providing study information for participants and obtaining their consent. Subsequently, baseline data and questionnaires were administered, followed by the preparation for injections and the blockwise randomization of the experimental conditions and injection sides of the back. The experimental phase was performed under three conditions (sham, local pain, remote pain) and was repeated three times for each participant in randomized order. For each condition, participants first reported their expectations regarding potential changes in lumbar movement control (“Do you think the injection will change your movement behavior in the back? Yes/No.”) as well as their fear of pain and fear of injection using 0–10 Numerical Rating Scales (NRS; 0 = no fear; 10 = greatest imaginable fear) to reflect the situational fear related to the experimental condition, which are widely used and validated for subjective pain- and fear-related ratings [29, 30]. Baseline lumbar movement control was then assessed using the lumbar movement control test battery (T1). Subsequently, the assigned injection was administered and the pain intensity evaluation commenced immediately afterwards, which was also assessed using a 0–10 NRS (0 = no pain; 10 = greatest imaginable pain). Lumbar movement control was then reassessed during the pain condition (T2), followed by completion the McGill Pain Questionnaire a widely used multidimensional measure of pain experience [31]. After a minimum of 15 min and once participants reported that pain has completely subsided, which was verified by the physician, lumbar movement control was assessed a third time (T3), marking the end of the respective experimental phase. Given the acute and transient nature of experimentally induced pain, a washout period of at least 20 min between injections was implemented to allow movement control and pain responses to return to baseline, in line with previous studies [32, 33]. No concomitant care or additional treatments were administered to participants. A schematic overview of the study procedure is provided in Fig. 1.

### Blinding and randomization

The research team comprised of a physiotherapist and a physician. The physiotherapist, a specialist in musculoskeletal therapy with extensive training in the test procedure, handled recruitment and lumbar movement control assessment. The physician provided participant information about the study, delivered the injections, and managed the randomized order of interventions per lottery method. The order of experimental conditions was randomized using a predefined set of all six possible sequences, each repeated equally and randomly assigned to participants. Both the intervention conditions (local pain, remote pain, sham) and the injection side (left/right) were randomized. Participants were blinded to the type of back injection but could, by necessity, distinguish whether the injection was given in the arm or the back. Pain rating was entered privately by participants on a handheld device and were only accessible to the physician, ensuring that the physiotherapist remained blinded to pain intensity. The physiotherapist conducting the lumbar movement control tests was not present during the injections, had no knowledge of the injection side or the pain rating and participants were instructed not to reveal the site of injection. To maintain blinding, adhesive bandages were applied by the physician to conceal all three potential injection sites during test performing. Lumbar movement control tests were organized into blocks based on starting positions and performed in a randomized sequence. The order of experimental conditions was block-randomized to control for potential sequence and carryover effects across testing conditions [34].

### Experimental pain induction

Each participant received three conditions including two injections of hypertonic saline (local and remote pain) and one injection of isotonic saline. The intramuscular injection of 1 mL hypertonic saline solution (5.85% NaCl) is well-established and safe for inducing acute low back pain [32, 33, 35]. As a sham injection, isotonic saline solution (NaCl 0.9%) was used.

**Fig. 2:**
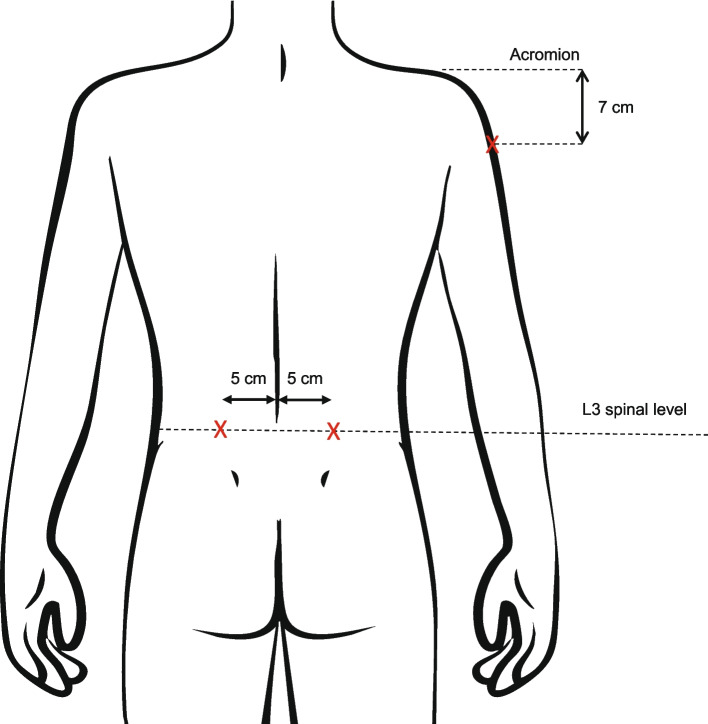
Injection sites at erector spinae muscle and deltoid muscle

The injection into the lumbar paraspinal muscle was performed using anatomical landmarks without ultrasound guidance. The procedure was conducted by an experienced clinician and followed previously described experimental pain protocols [33]. Hypertonic and isotonic saline injections into the paraspinal muscle were delivered with the participants positioned in prone, arms aligned alongside the torso. The spinous process of L3 was palpated, and the injection site marked 50 mm laterally (Fig. 2) [33, 36]. After cleaning the lower back twice with alcohol, a 40 mm needle was inserted perpendicular to the skin 5 mm above the mark to a depth of 30 mm [33, 37].

The deltoid muscle of the arm was selected as a remote pain site to avoid direct involvement of spinal muscles, thereby facilitating interpretation of trunk movement outcomes. For pain induction 0.5 ml hypertonic saline (5.85% NaCl) was injected into the deltoid muscle of the non-dominant arm [38, 39]. Participants were seated, with the arm supported at 60° abduction. The injection site, 7 cm below the acromion, was marked to avoid nerve and bursa injury (Fig. 2) [40, 41]. After cleaning twice with alcohol, a 40-mm needle was inserted perpendicularly, 5 mm deep [38].

Injections were administered according to a standardized protocol targeting the muscle belly. Injection depth was adjusted as necessary to account for individual differences in body composition, while the injected volume remained constant. Pain intensity was not used to calibrate the procedure.

### Lumbar movement control assessment

**Fig. 3:**
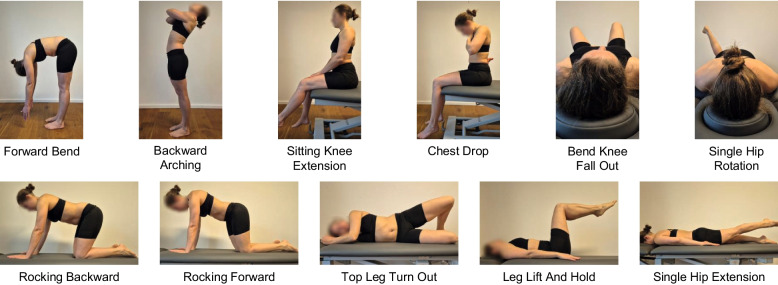
Lumbar movement control test battery. The tests include functional tasks performed in sitting, standing, and quadruped positions, targeting control of flexion, extension, and rotational movements. Each test requires participants to maintain controlled lumbar motion while minimizing compensatory movements of adjacent segments. Detailed instructions for each test are provided. Instructions: Forward Bend: Bend your upper body forwards and let your arms hang loosely. Backward Arching: Cross your arms in front of your chest and lean backwards with your upper body. Keep your knees straight. Sitting Knee Extension: Straighten your knee and keep your lumbar spine upright. Chest Drop: Cross your arms in front of your chest. Lower your gaze and round your cervical and thoracic spine. Stop the movement as soon as the lumbar spine starts to move. Bend Knee Fall Out: Put both feet on the therapy table with flexed knees and let the right/left knee tilt outwards. The foot remains in contact with the support. The pelvis and back should not move. Single Hip Rotation: Bend the right/left knee joint 90° and move the leg inwards and outwards. Your pelvis should not move at the same time. Rocking Backward: Move your buttocks towards your heels. Keep your hands on the mat and your back straight. Rocking Forward: Move your upper body forwards. Keep your knees and hands on the mat and your back straight. Top Leg Turn Out: Place your legs on top of each other and bend your hips 45° and your knees 90°. Raise your upper knee towards the ceiling, keeping your heels on top of each other. Keep your back and pelvis straight. Leg Lift And Hold: Put both feet on the therapy table with flexed knees and raise both legs simultaneously bent at the hips at 90° and hold the position briefly. Then put both feet back down at the same time. The lumbar spine should be in even contact with the support during the entire movement. Single Hip Extension: Please raise your right/left leg straight. The lumbar spine should remain straight

Lumbar movement control was assessed at three time points within each experimental condition: prior to the injection (T1), immediately after the injection (T2) and after 15 min (T3), when the pain had completely subsided. A direction-specific test battery was utilized to evaluate lumbar movement control [17], which includes standardized functional tasks performed in different positions (e. g. sitting, standing, quadruped), requiring participants to actively control lumbar spine movement while minimizing compensatory movements. To perform the tests correctly, various skills are required, such as controlled pelvic tilt, dissociation of lumbar and hip movement, and maintaining a neutral lumbar position during limb movements. These tasks are designed to challenge movement control across flexion, extension, and rotational components. A detailed overview of all tests, including instructions, is provided in Fig. 3. This test battery represents the optimal selection of tests with reported inter-rater reliability of at least substantial agreement (κ ≥ 0,61) [17], as derived from various established frameworks [13–15, 19, 42]. Additionally, item response theory analyses supported the structural validity of the test battery, indicating three underlying dimensions of lumbar movement control (flexion, extension and rotation/lateral flexion). Measurement precision was reported with standard errors of estimation ranging between 0.31–0.59 for flexion, 0.42–0.53 for extension, and 0.50–0.68 for rotation/lateral flexion control [17]. It comprises a total of 11 individual tests, four of which assess lumbar movement control in extension and flexion, and five in rotation, with two of these tests assessing two directions of movement simultaneously. The movement control tests were rated binary ("correct" = 0 or "incorrect" = 1). "Incorrect" was defined by premature or excessive movement [13, 14, 43]. For assessments performed on the right and left sides, tests for flexion or extension had to be performed “incorrectly” on both sides in order to arrive at this overall result. For the direction of movement rotation/lateral flexion, one “incorrect” side was sufficient to rate the test as “incorrect” overall [17]. Participants had two attempts to perform the test correctly if the instructions were misunderstood the first time. Figure 3 shows the complete test battery with corresponding instructions performing the tests. To evaluate lumbar movement control, the sum of the “incorrectly” performed tests for each participant was calculated, resulting in a scale of 0–11 (LMC Score). Since the research question focused on general changes in lumbar movement control, direction specificity was not evaluated separately. The results were documented in a standardized test protocol. Tests were randomized by starting position, and participants had two attempts if they misunderstood a test.

### Adverse event monitoring

Adverse events (e.g. prolonged pain, swelling, dizziness) were not systematically assessed but monitored through observation and participant self-report. No formal tools were used, as no side effects were expected. Participants were informed and could report symptoms at any time.

### Statistical analyses

An a priori power analysis was conducted assuming a mean effect size of f = 0.25 (α = 0.05, power = 0.9, three groups and three measurements, correlation among repeated measures = 0.5, nonsphericity correction ε = 1), which indicated a required minimum sample size of 36 participants. The calculations were performed using the software G*Power [44]. This medium effect size was chosen based on standard guidelines suggested by Cohen [45], as no prior studies were available to provide empirical estimates specific to this research question. Due to the possible dropout rate of approximately 20%, the number of participants was set at *n* = 44. No interim analyses were planned or conducted, and no stopping guidelines were established for this trial.

Descriptive statistics were calculated for all variables. Continuous variables (age, BMI, sitting time, pain intensity and duration) are reported as mean (SD), whereas questionnaire scores based on ordinal response scales (TSK, FPQ) are presented as median (Q1–Q3) and categorical variables as frequencies (n) and percentages (IPAQ-S Cat, occupational posture). Lumbar movement control was quantified using a sum score, calculated as the aggregate number of incorrectly performed tests. (0–11). The Shapiro–Wilk test showed non-normal distribution for LMC scores, pain intensity and duration requiring non-parametric tests for subsequent analyses.

Inferential statistical analyses were performed to detect changes in lumbar movement control scores within each condition across time points (T1–T3) and between conditions at each time point. Due to the deviations from normality, the Friedman test was selected as a robust, non-parametric approach suitable for repeated-measures data with small sample sizes [46]. Significant Friedman tests were followed by post-hoc pairwise comparisons using Bonferroni correction.

It was determined whether there were test subjects in the lumbar pain condition who demonstrated a minimum of 30% change (≥ 3 tests) from measurement time T1 to T2, to identify responders [47].

A further exploratory analysis was conducted to examine potential factors associated with lumbar movement control performance. The number of incorrect tests during the sham and pain_LB_ conditions at T2 was combined, resulting in 90 observations and used as a dependent variable (LMC_LB_). Potential predictors included sex, age, Body Mass Index (BMI), TSK, FPQ, IPAQ-S Cat, IPAQ-S Sit, fear of the injection and pain, occupational posture and expectations. Prior to model building, associations between these variables and LMC_LB_ were explored using Spearman's rho and Chi Square tests to identify potential predictors for lumbar movement control performance [48]. Variables demonstrating a significant correlation with LMC_LB_ were subsequently entered into the regression model. Due to the study aim, which was to explore whether pain influences lumbar movement control., mean pain intensity was included a priori. In total, five predictors were included for 90 observations, which is consistent with commonly suggested sample-size recommendations for regression models [46]. Given the count nature of the dependent variable, a Poisson regression model was initially considered. However, as overdispersion was detected (variance > mean), a negative binomial regression model was applied [49]. Model refinement was performed using backward elimination. This analysis was not intended to develop a predictive model but rather to explore potential associations with lumbar movement control performance.

All statistical analyses were carried out using IBM SPSS Statistics (version 29, Armonk, NY, USA). Statistical significance was set at *p <* 0.05. Since the dataset contained no missing values, no imputation procedures were applied.

## Results

### Population

**Fig. 4:**
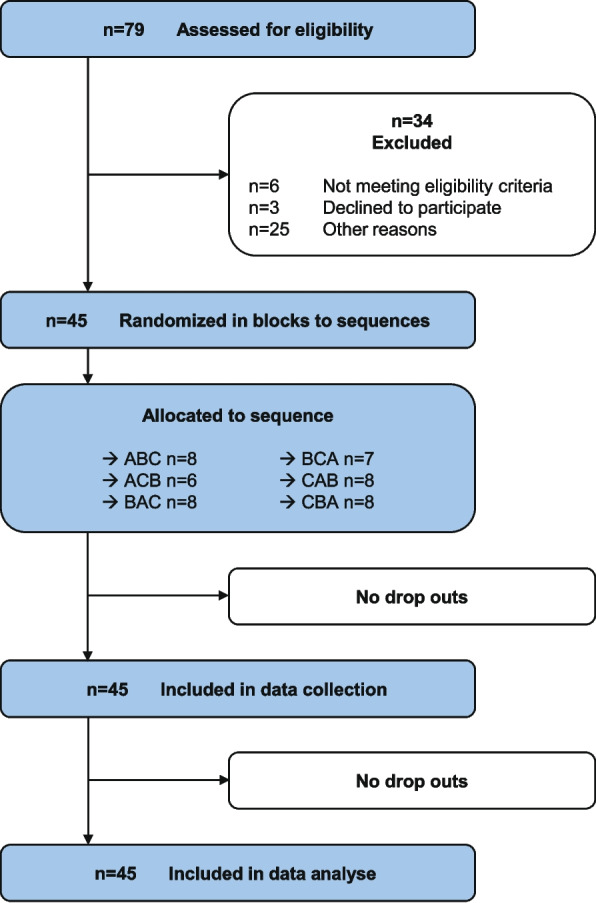
Modified CONSORT flow diagram for cross over trials (*n* = sample size; A = hypertonic saline back; B = hypertonic saline arm; C = isotonic saline back; CONSORT = Consolidated Standards of Reporting Trials). Other Reasons: unavailability: *n* = 9, illness: *n* = 4, non-appearance: *n* = 6, no suitable appointment possible: *n* = 6

Of the initial sample of 79 potential participants, six individuals were deemed ineligible, three withdrew due to an injection reluctance and 25 were excluded for various reasons (unavailability: *n* = 9, illness: *n* = 4, non-appearance: *n* = 6, no suitable appointment possible: *n* = 6). The final sample comprised 45 healthy participants, one more than planned, with no further drop-outs, unexpected side effects or any other adverse events (Fig. 4). The study sample had a mean age of 24 (SD 4.6) years and a Body Mass Index of 23.2 (SD 4.00). The IPAQ-S showed that one participant was inactive (2.2%), 22 (48,9%) were minimally active, 22 (48,9%) were active and the mean duration of daily sitting time was 6.11 h. The occupational posture was mainly sitting (88,9%, *n* = 40; standing 11,1%, *n* = 5) and the questionnaires showed Median (Q1-Q3) values for kinesiophobia (TSK 21 (16.0–22.5)) and fear of pain (FPQ-A 5 (3.0–7.0); FPQ-P 7 (4.5–10.0)). Further details are shown in Table 1.

**Table 1:** Characteristics of the study population

		**Total (** ***n*** ** = 45)**	**minimum**	**maximum**
Sex [% (*n*)]	m	48.90 (23)	-	-
	f	51.10 (22)	-	-
age [x̅ (SD)]		24.00 (4.64)	18	40
height in cm [x̅(SD)]		176.78 (9.20)	158	201
weight in kg [x̅(SD)]		73.04 (18.08)	50	140
BMI [x̅(SD)]		23.16 (4.03)	18.52	41.67
Occupational posture [% (*n*)]	sitting	88.9 (40)		
	standing	11.1 (5)		
TSK [M(Q1-Q3)]		21.00 (16.0–22.5)	11	36
FPQ_P [M(Q1-Q3)]		7.00 (4.5–10.0)	0	18
FPQ_A [M(Q1-Q3)]		5.00 (3.0–7.0)	0	13
IPAQ-S Cat [% (*n*)]	inactive minimally active HEPA active	2.20 (1) 48.90 (22) 48.90 (22)	-	-
IPAQ-S Sit in h [x̅(SD)]		6.11 (2.62)	2.00	12.00

Missing data (no answer) of four participants regarding the IPAQ-S Sit, *m* male, *f* female, *n* sample size, *x̅* mean, *SD* Standard deviation, *kg* kilogram, *M* median, *IQR* Interquartile range, *TSK* Tampa Scale of Kinesiophobia, *FPQ_A* Fear of Pain Questionnaire (Activity), *FPQ_P =* Fear of Pain Questionnaire (pain), *IPAQ-S Cat* Category of International Physical Activity Questionnaire Short form, *IPAQ-S Sit* sitting hours per day, *HEPA* Health enhancing physical activity, *h* hours

### Experimental acute pain

**Fig. 5:**
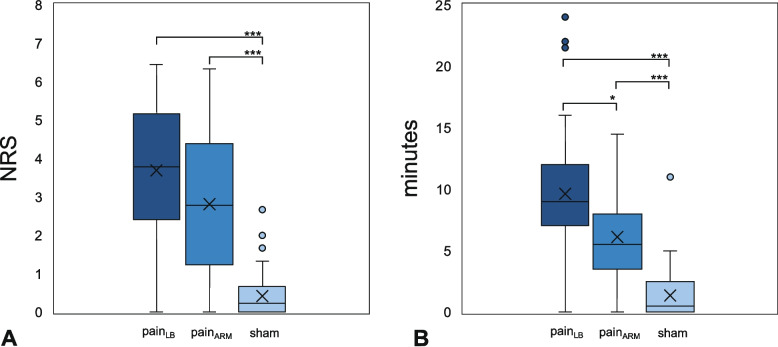
**A** Pain intensity on the Numeric Rating Scale; **B**: Pain duration in minutes after each injection Boxplots display the median (horizontal line), interquartile range (box; 25th–75th percentile), and mean values (cross). Whiskers indicate the largest/smallest values within 1.5 × IQR. Individual outliers beyond this range are shown as circles (NRS = Numeric Rating Scale; 0 = no pain; 10 = greatest imaginable pain) after each injection; (pain_LB_ = hypertonic saline back; pain_ARM_ = hypertonic saline arm; sham = isotonic saline back; *** *p <* 0.001; * *p =* 0,029)

In the sham condition, 82.4% of participants reported no or minimal pain (NRS 0–1/10; mean intensity 0.41 [SD 0.59]; mean duration 1.36 [SD 2.05] min) [50]. In the experimental conditions, mild to moderate pain (NRS 2–6/10) was reported by 88.9% (pain_LB_) and 71.1% (pain_ARM_) of participants [50]. Across the entire sample, mean pain intensities were 3.69 [SD 1.68] and 2.81 [SD 1.74], and mean durations were 9.62 [SD 4.96] and 6.12 [SD 3.86] minutes, respectively. All tests were completed within a maximum duration of 4.5 min. At the end of testing, mean pain intensity was 2.67 [SD 1.70] in the experimental back pain condition, 1.27 [SD 1.45] in the experimental arm pain condition, and 0.09 [SD 0.36] in the sham Friedman condition. tests revealed significant differences across conditions for both pain intensity (χ^2^(2) = 62.57, *p <* 0.001, W = 0.70) and duration (χ^2^(2) = 66.29, *p <* 0.001, W = 0.74). Post-hoc comparisons showed lower intensity and shorter duration of pain in the sham condition versus both experimental conditions (sham vs. pain_LB/ARM_: *p <* 0.001). While intensity did not differ between experimental sites (pain_LB_ vs. pain_ARM_: *p =* 0.105), duration was longer in the local condition (pain_LB_ vs. pain_ARM_: *p =* 0.029). Differences are illustrated in Fig. 5.

### Lumbar movement control

**Fig. 6:**
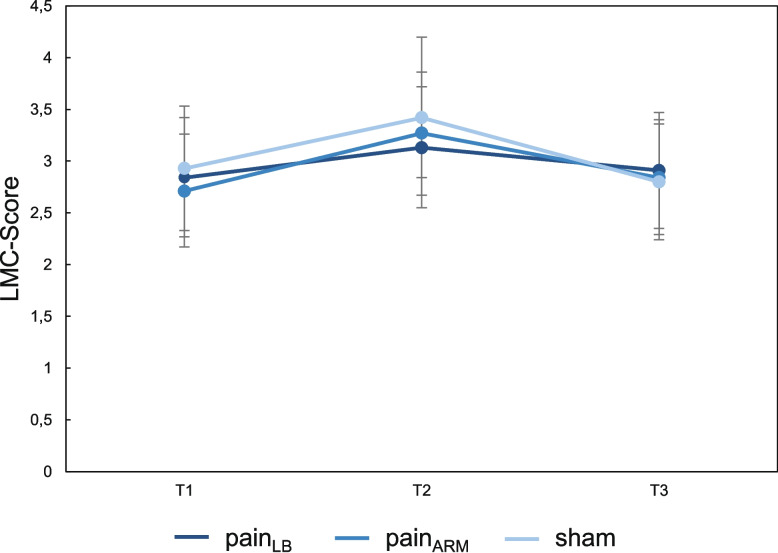
Number of incorrect tests (mean [95% CI]) at three different time points (T1 = before injection; T2 = after injection; T3 = after pain has subsided) under three conditions (sham = isotonic saline back; pain_LB_ = hypertonic saline back; pain_ARM_ = hypertonic saline arm; LMC-Score = number of incorrect lumbar movement control tests)

Analysis of LMC scores revealed no significant differences between conditions or across time points. Although the Friedman test indicated an overall effect (χ^2^(8) = 23.45; *p =* 0.003, W = 0.26), post-hoc comparisons showed no significant pairwise differences over time (pain_LB_: T1–T2/T2–T3/T1–T3: *p =* 1.00; pain_ARM_: *p =* 0.88/1.00/1.00; sham: *p =* 0.63/1.00/1.00) or between conditions (all *p =* 1.00). A transient increase in LMC scores was observed at T2 in all conditions, returning to baseline at T3. Mean scores (95% CI) were as follows: sham: T1 2.93 (2.33–3.53), T2 3.42 (2.84–4.00), T3 2.80 (2.24–3.36); pain_LB_: T1 2.84 (2.27–3.42), T2 3.13 (2.55–3.72), T3 2.91 (2.35–3.47); pain_ARM_: T1 2.71 (2.17–3.26), T2 3.27 (2.67–3.86), T3 2.84 (2.29–3.40). No responders were identified when defining response as a change of ≥ 3 lumbar movement control tests from T1 to T2 (*n* = 0). LMC scores for each condition and the corresponding pairwise comparisons are summarised in Table 2. Figure 6 illustrates means and 95% CIs over time.

**Table 2:** LMC scores for each condition and pairwise comparisons

Condition		LMC score [x̅ (95% CI)]		
		**T1**	**T2**	**T3**
Pain_LB_		2.84 (2.27–3.42)	3.13 (2.55–3.72)	2.91 (2.35–3.47)
Pain_ARM_		2.71 (2.17–3.26)	3.27 (2.67–3.86)	2.84 (2.29–3.40)
sham		2.93 (2.33–3.53)	3.42 (2.84–4.00)	2.80 (2.24–3.36)

Comparison		LMC score [MD (95% CI)]		*p*-value
Pain_LB_	T1-T2	−0.29 (−0.61 – 0.04)		1.00
	T1-T3	−0.07 (−0.51 – 0.37)		1.00
	T2-T3	0.22 (−0.26 – 0.70)		1.00
Pain_ARM_	T1-T2	−0.56 (−0.91 – 0.20)		0.88
	T1-T3	−0.13 (−0.44 – 0.18)		1.00
	T2-T3	0.42 (0.12–0.73)		1.00
Sham	T1-T2	−0.49 (−0.84 – −0.14)		0.63
	T1-T3	0.13 (−0.22 – 0.49)		1.00
	T2-T3	0.62 (0.31–0.93)		1.00
T1	pain_LB_-sham	−0.09 (−0.48 – 0.30)		1.00
	pain_ARM_-sham	−0.22 (−0.64 – 0.20)		1.00
	pain _LB_-pain _ARM_	0.13 (−0.29 – 0.56)		1.00
T2	pain_LB_-sham	−0.29 (−0.70 – 0.12)		1.00
	pain_ARM_-sham	−0.16 (−0.56 – 0.25)		1.00
	pain_LB_-pain_ARM_	−0.13 (−0.59 – 0.33)		1.00
T3	pain_LB_-sham	0.11 (−0.26 – 0.49)		1.00
	pain_ARM_-sham	0.04 (−0.32 – 0.41)		1.00
	pain_LB_-pain_ARM_	0.07 (−0.41 – 0.55)		1.00

*x̅* mean, *CI* Confidence interval, *LMC Score* number of incorrect lumbar movement control tests, *T1* before injection, *T2* after injection, *T3* after pain has subsided, *sham* isotonic saline back, *pain*_*LB*_ hypertonic saline back, *pain*_*ARM*_ hypertonic saline arm, *MD* mean difference, *p*-value = significance

Spearman correlation analyses and Chi Square tests identified four variables significantly associated with LMC_LB_ at T2 (BMI: r = 0.24, *p =* 0.02; IPAQ-S Cat: r = −0.36, *p <* 0.001; IPAQ-S Sit: r = 0.24, *p =* 0.03; sex: χ^2^ = 22.03, *p =* 0.01). Mean pain intensity was included unconditionally in the regression analyses. The initial model including BMI, IPAQ-S Cat, IPAQ-S Sit, sex, and mean pain intensity was significant (χ^2^(5) = 29.71, *p <* 0.001), but some variables contributed little to model quality. Following backward elimination, a model including BMI, IPAQ-S Cat, and IPAQ-S Sit provided the best fit (χ^2^(3) = 27.42, *p <* 0.001; AIC = 325.95, BIC = 337.98), which is shown in Table 3. BMI had a significant positive effect, with a 5% increase in incorrect tests per unit increase (β = 0.05, SE = 0.01, Exp(β) = 1.05, 95% CI [1.02–1.07], *p <* 0.001). Higher activity levels (IPAQ-S Cat) reduced incorrect tests by 29% (β = −0.34, SE = 0.11, Exp(β) = 0.71, 95% CI [0.58–0.88], *p =* 0.002). Daily sitting time (IPAQ-S Sit) showed a non-significant but notable effect, associated with a 4% increase in incorrect tests (β = 0.04, SE = 0.03, Exp(β) = 1.04, 95% CI [0.99–1.09], *p =* 0.15). Overall, higher BMI, lower activity, and longer sitting were linked to increased incorrect test performance.

**Table 3:** Regression Model at T2

T2	β	SE	Exp(β)	95% CI	*p*-value
BMI	0.05	0.01	1.05	1.02–1:07	< 0.001
IPAQ-S Cat	−0.34	0.11	0.71	0.59–0.88	0.002
IPAQ-S Sit	0.04	0.03	1.04	0.99–1.09	0.15

*T2* after injection, *BMI* Body Mass Index, *IPAQ-S Cat* Category of International Physical Activity Questionnaire Short form, *IPAQ-S Sit* sitting hours per day, *χ2(df)* Chi^2^(degree of freedom), *p-value* significance, *AIC* Akaike information criteria, *BIC* Bayes information criteria, *β* regression coefficient, *SE* Standard error, *Exp(β)* exponential coefficient, *CI* Confidence interval

## Discussion

The overall aim of this study was to isolate the immediate effect of experimentally induced pain on lumbar movement control. During induction and immediately after resolution, lumbar movement control remained stable, suggesting short-term robustness to transient nociceptive input. In an exploratory analysis, BMI, habitual physical activity and average daily sitting time were suggested as potential correlates of lumbar movement control performance, whereas pain intensity was not associated with the outcome.

In the present study, the hypertonic saline injection model was employed to investigate the acute nociceptive effects on lumbar movement control. This model enables the controlled and reversible induction of moderate deep somatic pain that closely mimics the sensory quality of clinical low back pain and has been extensively validated in experimental pain research [7, 51, 52]. The mean pain intensity achieved in this study (3.69 ± 1.2 NRS) was consistent with values typically reported in comparable studies (around 4–5/10) [35, 37, 53], supporting the reproducibility and validity of the model. Importantly, this approach allows for the isolation of nociceptive influences on motor control while minimizing confounding factors such as psychosocial or cognitive influences inherent to clinical pain populations [7]. The moderate pain intensity further ensured ethical acceptability while still evoking measurable nociceptive effects. Although no significant effects were observed for the primary outcome in the present study, previous research using the same model has demonstrated pain-induced alterations in motor unit recruitment and postural control [7], underscoring its sensitivity for detecting pain-related neuromuscular adaptations. Taken together, these characteristics make the hypertonic saline model a suitable and robust approach for experimentally examining acute nociceptive effects on lumbar movement control.

To date, the impact of acute, first-episode low back pain on movement control has remained largely unexplored, as existing research often conflates acute and recurrent pain episodes [54–56]. The current study contributes to this gap by suggesting that short-lasting experimentally induced nociceptive input, as elicited by saline injection, may not substantially alter lumbar movement control under the conditions tested. Notably, assuming a relevant change in the number of incorrect tests (30%), no participant exhibited a substantial response before and after the lumbar injection. This consistency contrasts with the highly individual and variable response to experimental pain at other motor levels [32, 57–60] and may reflect adaptive neuromuscular strategies that preserve functional movement despite nociceptive input [57, 59, 61].

The question of whether pain drives movement control impairments or whether these impairments arise independently of pain must be viewed in a differentiated way. While some studies posit pain as a primary driver of movement control deficits [10, 61, 62], others suggest that such impairments may precede pain onset or exist independently of it [54, 59, 63]. Regression analyses revealed no significant association between acute pain intensity and the number of incorrect lumbar movement control tests. Similar findings were reported by Bauer et al., who examined patients with varying back pain intensity using tests included in the current battery [62]. They observed that higher pain intensity was associated with increased movement variability, suggesting an effect on movement strategies rather than direct impairment of movement control. Similarly, another study [54] investigated the relationship between movement control impairments and pain intensity over 12 months in patients with acute low back pain using a comparable, though less extensive, test battery, and found no significant correlation. A broader perspective is offered by a systematic review and meta-analysis, which identified a small effect size linking pain intensity with movement control. However, the authors concluded that pain intensity alone is unlikely to be a primary determinant of movement alterations [63]. Collectively, these findings suggest that subjective longitudinal assessment of lumbar movement control may remain unchanged following the induction of brief local or remote nociceptive input under the experimental conditions tested.

While the current study was unable to identify any impact of acute experimental pain on the lumbar movement control test battery, long-term factors such as pain severity, location, and duration, may play a significant role [10, 64]. Changes in motor cortical organization and output are linked to low back pain severity and location, with cortical alterations producing smaller, overlapping muscle representation fields that impair individual muscle control [10, 64]. These neuromuscular inefficiencies lead to altered movement patterns and increased lumbar variability [10, 64]. When considered in conjunction with the findings of the present study, it becomes evident that the persistence of pain exerts a more significant influence on the development of lumbar movement control disorders than the pain intensity.

If nociception alone does not primarily drive movement control impairments, at least in the acute stage, other factors may contribute to the differences observed between healthy individuals and patients with chronic low back pain. In the present study, BMI, daily sitting time, and activity level were associated with lumbar movement control performance, suggesting that movement control may be influenced by multiple interacting factors. This is consistent with findings by Mikkonen et al. [65], who reported that ageing and higher BMI accounted for movement control impairments more effectively than chronic pain itself, supporting the notion that demographic and behavioral characteristics can substantially shape lumbar movement control. Together with compensatory motor mechanisms due to pain, may contribute to maladaptive learning over time. This is in line with evidence linking chronic pain to cortical reorganization and motor learning adaptions [10, 64, 66]. Habitual movement behaviors, such as prolonged sitting or repetitive movements, may further influence lumbar movement control over time, potentially contributing to maladaptive motor strategies and possible chronic pain development [14, 15, 54].

This study possesses several methodological strengths that enhance its validity. Blinding minimized observer bias, while randomization and a crossover design ensured balanced group allocation. Repeated measurements before, during, and after pain allowed for a detailed temporal analysis, and the recruitment of a homogeneous, healthy cohort reduced confounding variability, reinforcing the reliability of the findings. However, certain limitations must be acknowledged. Compared with experimentally induced pain, clinical pain is characterized by greater perceived threat, heightened protective strategies, and more pronounced motor alterations [67, 68]. While the hypertonic saline model allows for controlled and reversible induction of moderate deep somatic pain resembling low back pain, the induced pain is not movement-dependent, which may limit direct comparability to clinical pain experiences [37, 51]. Although participants were instructed not to disclose the site of injection they received, complete blinding of participants could not be fully ensured due to the perceptible difference in pain between hypertonic and isotonic injections. This may have influenced participant expectations or responses, and should be considered when interpreting the results. Additionally, the restriction to a young, healthy cohort limits transferability to individuals with low back pain. Although the psychometric properties of the lumbar movement control test battery have been evaluated using item response theory, some tests may have limited sensitivity in this population, as some tasks might have been too simple to detect subtle movement control deficits [17]. Moreover, direct comparisons with instrumented kinematic assessments (e.g., motion capture) are currently lacking. No EMG or kinematic analyses were performed, so subtle adaptations or protective strategies, such as reduced movement speed or amplitude, could not be captured. Furthermore, the assessment relied on visual observation by a single trained therapist, which may introduce some degree of interpretive variability. However, previous studies have reported substantial inter-rater reliability for the lumbar movement control tests when performed by trained physiotherapists in controlled settings [17, 42, 43]. Nevertheless, the short duration of experimentally induced pain precluded the use of a second assessor, so a certain degree of measurement imprecision cannot be completely excluded.

In conclusion, lumbar movement control in young, healthy adults remained stable during experimentally induced acute nociceptive pain and immediately after its resolution, indicating short-term resilience to transient nociceptive input. These findings suggest that brief experimental pain alone is insufficient to induce measurable lumbar movement control impairments in this population. Future research should examine more heterogeneous and clinical cohorts and incorporate complementary, objective measures to gain deeper mechanistic insights into the role of pain in movement control disorders. In addition, experimental models inducing more sustained nociceptive input (e.g., nerve growth factor injections) may help to better understand whether longer-lasting pain leads to measurable alterations in lumbar movement control.

## Data Availability

The datasets used and/or analyzed during the current study are available from the corresponding author on reasonable request.
